# Nutritional Interventions in Type 1 Diabetes: Boosting Residual GLP-1 Responses—Is It an Option?

**DOI:** 10.3390/nu18040564

**Published:** 2026-02-09

**Authors:** Maria Grammatiki, Xanthippi Tsekmekidou, Theocharis Koufakis, Kalliopi Kotsa

**Affiliations:** 1 Division of Endocrinology and Metabolism and Diabetes Center, First Department of Internal Medicine, School of Medicine, Aristotle University of Thessaloniki, AHEPA University Hospital, 54636 Thessaloniki, PC, Greece; grammatikimaria@gmail.com (M.G.); xanthippitsekmekidou@gmail.com (X.T.); 2 Second Propedeutic Department of Internal Medicine, Hippokration Hospital, School of Medicine, Aristotle University of Thessaloniki, 54636 Thessaloniki, PC, Greece; thkoyfak@auth.gr

**Keywords:** type 1 diabetes mellitus, incretin hormones, GLP-1, glucagon-like peptide-1, nutrition, Mediterranean diet, short-chain fatty acids, glycemic index

## Abstract

Type 1 diabetes (T1D) is characterized by autoimmune beta-cell destruction and lifelong insulin dependence, yet early-stage disease (Stages 1–2) retains residual beta-cell function that may still respond to incretin signaling. Incretin hormones—mainly glucagon-like peptide-1 (GLP-1) and glucose-dependent insulinotropic polypeptide (GIP)—enhance postprandial insulin secretion and suppress glucagon, and GLP-1 also exhibits beta-cell protective effects in preclinical models. Although the incretin effect is markedly reduced in established T1D, intestinal GLP-1 secretion is largely preserved, creating a mechanistic rationale for strategies that increase endogenous GLP-1 during the “residual function” window. This narrative review summarizes dietary and lifestyle interventions that may enhance endogenous GLP-1 responses and discusses their potential role as adjuncts to insulin therapy, particularly when combined with emerging beta-cell-preserving immunomodulatory approaches that may prolong early disease stages. Mechanistically, high-fiber diets may increase GLP-1 via microbiota-derived short-chain fatty acids acting on L-cell receptors; low-glycemic index carbohydrates may favor distal nutrient delivery and a GLP-1-dominant incretin profile; and Mediterranean dietary patterns may promote GLP-1 secretion through unsaturated fatty acids, fiber, and polyphenols, including potential DPP-4-modulating effects. This narrative review examines nutrition and lifestyle interventions modulating residual incretins to elongate early T1D stages and enhance glycemic control as insulin adjuncts, per *Nutrients*’ Special Issue. Available evidence is strongest in non-T1D populations, with limited T1D-specific trials, highlighting the need for stage-targeted studies incorporating GLP-1 dynamics, C-peptide, glycemic variability, and microbiome outcomes.

## 1. Introduction

Type 1 diabetes (T1D) is a chronic autoimmune disease characterized by progressive pancreatic beta-cell destruction, resulting in absolute insulin deficiency and lifelong dependence on exogenous insulin therapy. Despite major advances in insulin analogs, pumps, and continuous glucose monitoring, many individuals with T1D continue to experience substantial glycemic variability and hypoglycemia risk, supporting the need for adjunctive, physiology-based strategies beyond insulin replacement alone.

Incretin hormones—primarily glucagon-like peptide-1 (GLP-1) and glucose-dependent insulinotropic polypeptide (GIP)—potentiate nutrient-stimulated insulin secretion and contribute substantially to postprandial glucose regulation in individuals without diabetes [1]. Beyond its acute insulinotropic and glucagonostatic actions, GLP-1 has been associated with beta-cell protective effects in preclinical models, including improved beta-cell survival and proliferative signaling, which has fueled interest in incretin-targeted approaches as disease modifiers rather than glucose-lowering tools alone [2]. In established T1D, however, the classical incretin effect is markedly diminished due to loss of functional beta-cell mass, although GLP-1 secretion from intestinal L-cells is largely preserved, given its pancreatic beta-cell-independent origin. Postprandial GLP-1 levels in T1D are comparable to or slightly reduced compared to healthy controls, with exaggerated glucagon responses persisting despite GLP-1 elevation [3,4,5].

A key conceptual shift is that T1D is increasingly viewed as a continuum with identifiable presymptomatic stages prior to clinical diagnosis (Table 1). In the widely adopted staging framework, Stage 1 is defined by the presence of two or more islet autoantibodies with normoglycemia, Stage 2 is defined by autoimmunity with dysglycemia (still without symptoms), and Stage 3 is defined by symptomatic, clinically diagnosed diabetes [6]. These early stages retain variable residual beta-cell function, creating a therapeutic window in which endogenous insulin secretion remains measurable and potentially responsive to enteroinsular signaling, including incretin-mediated pathways.

This window has become even more clinically relevant with the emergence of disease-modifying immunotherapy. Teplizumab (anti-CD3) has received regulatory approval to delay progression to Stage 3 T1D in eligible individuals with Stage 2 disease, highlighting that preservation of beta-cell function is an achievable clinical goal and extending the time during which adjunctive metabolic strategies might have a greater impact [7]. In this context, nutritional interventions represent a particularly attractive, scalable approach: they can modulate endogenous incretin secretion (especially GLP-1) through defined mechanisms such as fermentation of dietary fibers into short-chain fatty acids (SCFAs), altered intestinal nutrient delivery with low-glycemic index (GI) carbohydrates, and Mediterranean dietary patterns rich in unsaturated fats, polyphenols, and fiber without added hypoglycemia risk [8,9].

Accordingly, this narrative review evaluates dietary and lifestyle interventions that may enhance endogenous GLP-1 responses and discusses their potential role as adjuncts to insulin therapy across the T1D disease continuum, with emphasis on early-stage (Stages 1–2) disease and on translational opportunities in the era of beta-cell-preserving immunomodulation. We summarize mechanistic pathways, appraise the available clinical evidence (including indirect evidence from type 2 diabetes (T2D) and prediabetes where relevant), and highlight key research gaps for future stage-targeted trials that incorporate incretin dynamics and beta-cell function outcomes.

## 2. Methods

This narrative review aimed to synthesize current evidence on nutritional and lifestyle strategies that may enhance endogenous incretin activity—particularly GLP-1—in type 1 diabetes (T1D), with emphasis on early-stage disease where residual beta-cell function may persist. A literature search was conducted in PubMed/MEDLINE and Google Scholar using combinations of keywords and related terms including “type 1 diabetes”, “stage 1”, “stage 2”, “presymptomatic”, “incretin”, “GLP-1”, “GIP”, “incretin effect”, “diet”, “nutrition”, “dietary fiber”, “β-glucan”, “inulin”, “prebiotic”, “short-chain fatty acids”, “glycemic index”, “isomaltulose”, and “Mediterranean diet”. Additional records were identified by screening the reference lists of relevant reviews and key primary studies, prioritizing clinical trials, systematic reviews/meta-analyses, mechanistic human studies, and high-relevance preclinical work that informed biological plausibility. The final selection of evidence was organized thematically (fiber/SCFA pathways; low-GI carbohydrate effects on distal L-cell stimulation; Mediterranean diet (MedDiet) components and putative DPP-4–related effects), and the findings were discussed with explicit attention to translational relevance and current evidence gaps specific to T1D.

## 3. Results: Dietary Interventions Enhancing Endogenous GLP-1 Secretion and Actions

Nutrition strategies targeting enteroendocrine L-cell stimulation could represent the cornerstone of medical nutrition therapy and incretin modulation in early T1D, leveraging preserved GLP-1 secretion independent of beta-cell loss. High soluble fiber intake, low-GI (GI < 55) carbohydrates, and Mediterranean dietary patterns have shown potential to enhance postprandial GLP-1 responses through distinct L-cell stimulation mechanisms, with evidence from preclinical models, healthy individuals, and T2D cohorts suggesting improved glycemic control that may translate to preserved C-peptide and reduced variability in early T1D Stages 1–3.

### 3.1. High-Fiber Diets: Short-Chain Fatty Acids Mediated L-Cell Activation

A high-fiber dietary approach emphasizes nutrient-dense whole plant foods, including berries and skin-on apples, broccoli alongside skin-on sweet potatoes and dark leafy greens, whole grains such as oats and barley, and legumes like lentils, beans, and peas, supplemented by chia seeds and almonds to achieve target intakes that support digestive regularity and microbiota diversity through balanced meals.

Indigestible soluble fibers—including β-glucans from oats, psyllium husks, and inulin from chicory—undergo colonic fermentation by microbiota, producing SCFAs. Acetate, propionate, and butyrate constitute over 80% of total SCFAs produced, with chain length (C2–C5) dictating receptor affinity [10].

SCFAs can trigger cell-specific signaling cascades by activation of the G-protein-coupled free fatty acid receptors FFAR2 (GPR43) and FFAR3 (GPR41) [11]. Both receptors are located in colonic L cells [12,13], suggesting that SCFAs may use this pathway to modulate L-cell function, initiating calcium influx and GLP-1 granule release independent of pancreatic beta cells. Experimental data on a transgenic mouse model and isolated perfused rat colon supported this idea some years ago [14,15].

Evidence from clinical studies related to this concept is still inconsistent. B-glucan is a type of soluble dietary fiber found in oats and barley with known cholesterol-lowering benefits and an excellent SCFA source candidate to study. In a randomized double-blind, crossover design study in healthy normal-weight subjects, oat β-glucan consumption was reported to lower appetite and beneficially modulate postprandial glycemia, but it did not increase plasma GLP-1 secretion [16]. Similar results, regarding oat β-glucan consumption, have been reported in diabetic populations. In thirty-seven T2D subjects, 12 weeks of daily oat β-glucan consumption (5 g/day) as part of a regular diet had a beneficial effect on the metabolic control of T2D subjects, improving glycemic control and triglyceride levels while GLP-1 levels decreased significantly [17]. A recent meta-analysis of the existing data concludes that the current evidence provides a very good indication for reductions in fasting glucose and less of an indication for reductions in HbA1c, 2h-PG, fasting insulin, and HOMA-IR in T2D populations, confirming the dose–response hypothesis of β-glucan concerning glycemic response. Data on GLP-1 responses remain limited, highlighting the inconsistency regarding human and animal studies [18]. Premeal and post-meal blood glucose levels were lowest for breakfast, lunch, and overall, when adolescents with T1D consumed oat flakes containing 6 g/day of β-glucan for one week compared to oat flakes containing 3 g/day of the β-glucan or a standard diet. Oat flakes containing 6 g/day β-glucan were also related to significantly lowering glycemic variability-related parameters, but the study reported no results on GLP-1 levels [19].

Another is inulin. Inulin-type fructans rank among the best-studied prebiotic fibers for blood glucose regulation. As fermentable fibers, they slow gastric emptying and glucose absorption in the gut, blunting postprandial spikes. Moreover, basic science studies have shown that inulin accelerates GLP-1 release primarily through colonic fermentation by gut microbiota, producing short-chain fatty acids that trigger the SCFA-L-cell axis.

Animal studies are in agreement with these results. In a recent study, STZ-induced diabetic rats were fed a high-fat diet, and treatment with inulin-type fructans supplementation significantly improved the diabetic phenotype through gut microbiota modulation [20]. Inulin treatment elevated serum GLP-1 levels (*p* < 0.01), improved OGTT AUC by 25.8%, and restored beneficial gut bacteria. These findings demonstrate inulin’s capacity to enhance endogenous GLP-1 responses and improve glycemic control in experimental diabetes models via microbiota-dependent pathways.

Human studies underline the different results in different populations. In regard to healthy participants, Kietsiriroje et al. (2018) reported significant postprandial GLP-1 increases (AUC *p* = 0.003) in 26 healthy young men after 8 weeks of 10 g/day inulin plus phytosterols in soymilk [21]. Moreover, Steinert et al. (2024) observed comparable early-phase GLP-1 secretion at 30 min postprandially in post-RYGB patients receiving acute inulin loads versus maltodextrin, accompanied by faster colonic fermentation [22]. In contrast to the above results, a similar study in 29 T2D participants reported no significant changes in postprandial GLP-1 levels after 12 weeks of 6 g daily inulin-type fructans supplementation. This negative trial highlights inconsistent ITF effects in T2D populations [23]. Wang L et al. conducted a GRADE-assessed systematic review and meta-analysis of 33 RCTs involving 1346 participants, primarily prediabetes and T2DM patients. Inulin-type fructans (ITF) supplementation significantly reduced fasting blood glucose (WMD: −0.49 mmol/L), HbA1c (−0.58%), fasting insulin (−1.75 µU/mL), and HOMA-IR (−0.69), with optimal effects at ≥10 g/day for ≥6 weeks. Subgroup analyses showed stronger benefits were influenced by participant sex, ITF type, and intake method, supporting ITF as adjuvant therapy for glycemic management in prediabetes/T2D [24].

Limited data on T1D patients exist. In a randomized controlled trial, Ho et al. randomized 43 children with T1D to 8 g/day oligofructose-enriched inulin or maltodextrin for 12 weeks (38 completers), demonstrating significant stimulated C-peptide increases (*p* = 0.029) in the prebiotic group, alongside improved intestinal permeability trends (*p* = 0.076). No significant HbA1c changes occurred. Interestingly, the study did not report GLP-1 measurements despite the protocol listing them as secondary outcomes [25].

### 3.2. Low-Glycemic Index (GI < 55) Carbohydrates

The GI is defined as the incremental area under the blood glucose response curve (iAUC) elicited by ingesting a portion of food containing 50 g of available carbohydrates, expressed as a percentage of the response to an equivalent amount of reference carbohydrates (pure glucose or white bread) in healthy subjects after an overnight fast [26]. Low-glycemic index carbohydrates (GI < 55), such as legumes, whole grains, barley, and non-starchy vegetables, are foods that digest and absorb slowly (GI < 55), minimizing rapid blood glucose spikes compared to high-GI options (>70) like white bread or potatoes.

Low-GI carbohydrates bypass proximal K-cells and minimize rapid proximal nutrient exposure that triggers duodenal K-cell GIP secretion. Instead, they favor gradual distal delivery to ileal/colonic L-cells that are responsible for GLP-1 release [27]. High-GI carbohydrates, conversely, cause rapid duodenal glucose spikes that predominantly activate K-cells that are responsible for GIP release, with limited distal L-cell engagement. Moreover, low-GI diets also foster beneficial gut bacteria that produce SCFAs, which further support GLP-1 production and overall metabolic health [27]. Therefore, in light of the above, low-GI carbohydrates have been connected with low postprandial endogenous GIP levels and increased GLP-1 concentrations.

Isomaltulose (Palatinose) provides an elegant model to isolate the physiological effects of glycemic index on incretin biology because it is structurally similar to sucrose but differs in the rate of small-intestinal digestion. Isomaltulose and sucrose are both glucose–fructose disaccharides, but sucrose has an α1,2-glycosidic bond that is rapidly hydrolyzed by intestinal glucosidases, whereas isomaltulose has an α1,6-glycosidic bond that is cleaved much more slowly, leading to delayed yet complete absorption in the small intestine, as confirmed in ileostomy patients. This complete small-intestinal uptake ensures that differences in metabolic and hormonal responses can be attributed to GI per se, rather than to colonic fermentation or microbiota-mediated effects that would arise if carbohydrates reached the large intestine. Compared with sucrose, isomaltulose produces a slower, more gradual rise in blood glucose and insulin with lower peak concentrations, indicating improved postprandial glucose tolerance. Using this pair of isoenergetic, compositionally similar sugars, studies have demonstrated that lowering GI with isomaltulose modifies intestinal hormone responses—including incretins—alongside effects on insulin sensitivity, hepatic lipid accumulation, fatty liver risk, and inflammatory markers, thereby providing a mechanistically clean tool to dissect how high- versus low-GI carbohydrates shape GLP-1 and GIP secretion in humans [28].

Indeed, in normal volunteers, oral isomaltulose (typically 50 g) produced a slower, lower rise in plasma glucose and insulin compared with an isocaloric sucrose load [29]. Regarding incretin secretion, isomaltulose versus sucrose led to significantly lower early postprandial plasma GIP (15–90 min), while total and active GLP 1 concentrations were significantly higher at 90 min, and GLP 1 AUC (60–120 min) was greater with isomaltulose, which is consistent with delayed distal L cell stimulation [29].

Similarly, in patients with T2D, acute isomaltulose ingestion elicited significantly lower and more gradual postprandial glucose and insulin excursions than sucrose, despite identical carbohydrate loads. Crucially, incretin profiles diverged: GIP concentrations were consistently lower after isomaltulose, whereas GLP 1 levels and GLP 1 AUC in the later postprandial phase were higher compared with sucrose [30].

Isomaltulose-based comparisons with sucrose in human studies illustrate that simply slowing small-intestinal carbohydrate digestion—without altering macronutrient composition or colonic delivery—shifts the enteroendocrine response toward a lower glycemic profile with distinct incretin dynamics, providing strong evidence that GIP and GLP-1 secretion are modulated by carbohydrates’ glycemic index.

### 3.3. Mediterranean Diet

The MedDiet is a traditional eating pattern originating from countries bordering the Mediterranean Sea, such as Greece, Italy, and Spain, characterized by high consumption of plant-based foods including fruits, vegetables, whole grains, legumes, nuts, and seeds; olive oil as the primary fat source; moderate intake of fish, poultry, dairy (especially yogurt and cheese), and red wine with meals; and low consumption of red and processed meats, sweets, and refined sugars. This dietary model emphasizes minimally processed, seasonal foods rich in monounsaturated and polyunsaturated fats, fiber, antioxidants, and polyphenols, promoting synergistic health benefits through its holistic composition rather than isolated nutrients. Extensively studied since the mid-20th century, the MedDiet is associated with reduced risks of cardiovascular disease, type 2 diabetes, and metabolic disorders, underpinning its relevance in nutritional interventions (Table 2).

The boosting effects of MedDiet on GLP-1 secretion and action are supported by well-defined mechanisms. While GLP-1 secretion is triggered by a variety of nutrients (carbohydrates, lipids, and proteins), animal and human studies have underlined the importance of the chain length and degree of saturation of fatty acids FA in GLP-1 secretion [31,32]. In this sense, polyunsaturated fatty acids (from fatty fish, nuts, and seeds) and monounsaturated fatty acids (from extra-virgin olive oil) have been shown to be more effective GLP-1 secretagogues than saturated fats [31,32]. Most likely, polyunsaturated fatty acids present in MedDiet bind to and activate G protein-coupled receptors, such as GPR120, thereby increasing GLP-1 production from enteroendocrine L cells [33].

MedDiet, as mentioned before, is rich in polyphenols. Polyphenols activate cAMP signaling pathways in L-cells, enhancing proglucagon gene expression and GLP-1 biosynthesis [34]. They also exhibit DPP-4 inhibitory effects in vitro and in vivo, reducing degradation of active GLP-1 (7–36) amide to extend its half-life and incretin action [34].

Finally, high fiber from fruits, vegetables, legumes, and whole grains supports gut health, indirectly enhancing L-cell stimulation via SCFAs producing microbiota that stimulate FFAR2/FFAR3 signaling, with mechanisms that were mentioned earlier. Oral extra-virgin olive oil (EVOO) supplementation in NOD mice slowed gastric emptying, reduced pancreatic insulitis, increased the Bacteroidetes/Firmicutes ratio with more SCFA-producing bacteria (e.g., Lachnoclostridium), and delayed type 1 diabetes onset—suggesting microbiota-targeted nutrition as a strategy for early intervention [35]. A 2025 study showed that extra-virgin olive oil boosts Akkermansia muciniphila abundance in the human gut and enhances GLP-1 secretion from enteroendocrine L-cells via SCFA signaling. Although direct T1D trials are lacking, this diet–microbe–incretin axis suggests microbiome-targeted strategies to enhance residual incretin function in type 1 diabetes [36].

Although there are limited data for patients with T1D, the above has been confirmed by studies in patients with impaired glucose tolerance and T2D [32,37,38,39]. Indeed, studies in patients with impaired fasting glucose consistently show that taking extra-virgin olive oil (rich in polyphenols) increases blood insulin and GLP-1 levels, especially in the postprandial phase. Favorable effects in blood glucose levels were also accompanied by a reduction in DPP-4 levels [37,38]. Moreover, in a randomized controlled crossover trial involving 12 overweight or obese patients with T2D, isocaloric Mediterranean meals significantly increased the GLP-1 area under the curve over 210 min (*p* < 0.022) and oxyntomodulin levels (*p* < 0.023) compared with high-fiber vegetarian meals. The GLP-1 response exhibited a biphasic pattern, with a notably higher delayed peak at 150 min (*p* < 0.05), despite equivalent fiber content between the two diets [32]. Similarly, an acute study in patients with T2D showed that an olive oil-supplemented MedDiet prevented hyperglycemia-induced endothelial dysfunction and restored GLP-1′s protective signaling [39]. In a small trial of 11 adults with T1D on insulin pump therapy, researchers compared postprandial responses to three high-glycemic-index meals that differed only in fat content and quality. Extra-virgin olive oil supplementation enhanced GLP-1 and GIP secretion compared to a high-saturated-fat control meal, with these incretin elevations correlating to lower postprandial glucose excursions and modulated gastric responses [40].

These mechanisms converge in comprehensive dietary patterns. Kahleova et al. (2024) conducted the largest T1D dietary RCT to date, randomizing 35 adults to 12 weeks of a low-fat vegan diet versus a portion-controlled diet [41]. The intervention group achieved dramatic reductions in total daily insulin (−12.1 units, *p* = 0.007), increased insulin sensitivity (6.6 g of carbohydrate per unit of insulin on average (*p* = 0.002)), and improved HbA1c (−0.8%) without compromising glycemic control. These outcomes likely reflect synergistic GLP-1 enhancement through high soluble fiber (SCFA production), low glycemic load (distal L-cell preference), and minimal saturated fat—directly validating the convergent mechanisms reviewed here.

## 4. Conclusions and Limitations

**Figure 1:**
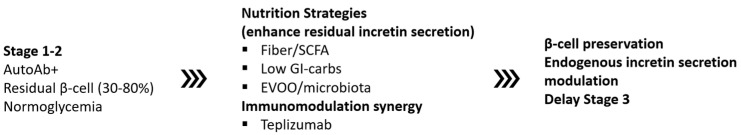
The T1D incretin window framework.

Dietary strategies that enhance endogenous GLP-1 secretion represent a plausible, low-cost adjunct to insulin therapy in T1D, particularly during early-stage disease when residual beta-cell function may still permit physiologically meaningful incretin effects. The approaches with the most coherent mechanistic rationale include increasing soluble/fermentable fiber (via microbiota-derived SCFAs and L-cell stimulation), prioritizing low-GI carbohydrates to promote distal nutrient delivery and a GLP-1-dominant enteroendocrine response, and adopting Mediterranean dietary patterns rich in unsaturated fats, polyphenols, and fiber (Box 1). The clinical relevance of this “incretin window” may expand as disease-modifying therapies—such as teplizumab, which is shown to delay progression to clinical (Stage 3) T1D in high-risk individuals—extend the period of residual beta-cell function (Figure 1).

Box 1 **Clinical recommendations for dietary strategies that may enhance endogenous GLP-1 signaling in T1D.**

- Target population: Prioritize implementation in early-stage T1D (Stages 1–2) and early Stage 3 when residual beta-cell function is more likely to permit clinically meaningful incretin-mediated effects.
- Preferred overall pattern: Use a Mediterranean-style eating pattern emphasizing minimally processed plant foods, extra-virgin olive oil, nuts/legumes, and fish, which is consistent with mechanisms reviewed for GLP-1 support (unsaturated fats, polyphenols, and fiber synergy).
- Fiber/SCFA approach (high priority): Increase soluble/fermentable fibers (e.g., β-glucans; inulin-type fructans) gradually to support SCFA production and L-cell stimulation; titrate to tolerance to minimize gastrointestinal symptoms and improve adherence.
- Low-GI carbohydrate approach (high priority): Prefer low-glycemic index carbohydrate sources (e.g., legumes, intact whole grains) to slow proximal absorption and favor distal nutrient delivery, supporting a GLP-1-dominant incretin profile in mechanistic models.
- Clinical implementation with insulin: Introduce changes with CGM-guided evaluation of postprandial profiles and anticipate shifts toward later postprandial excursions; adjust insulin timing/dose as needed to reduce risk of delayed hypoglycemia.
- What to monitor (practical endpoints): CGM time-in-range/time-below-range, postprandial peak and late postprandial patterns, glycemic variability, total daily insulin dose, and (where feasible in early-stage settings) C-peptide trends.

However, translation into evidence-based recommendations remains constrained by important limitations. First, much of the supportive human evidence for diet-induced GLP-1 augmentation derives from studies in healthy participants, prediabetes, and T2D, while T1D-specific randomized trials are scarce and often do not measure incretin endpoints directly. Second, existing T1D dietary trials are heterogeneous in intervention type, duration, and outcomes; for example, prebiotic oligofructose-enriched inulin improved stimulated C-peptide in a pediatric randomized trial, but GLP-1 outcomes were not reported, limiting mechanistic inference. Third, inter-individual variability (baseline microbiome composition, degree of dysglycemia, insulin regimen/technology, and stage of autoimmunity) likely modifies response, complicating generalization from small studies and short interventions.

Key research gaps, therefore, include stage-targeted trials that explicitly test whether dietary incretin modulation can preserve beta-cell function or improve clinically meaningful outcomes in T1D. Priority designs should enroll participants across Stages 1–3 with standardized phenotyping (autoantibodies, C-peptide, and CGM metrics), include serial measurements of active/total GLP-1 (and ideally GIP and glucagon), and integrate microbiome and metabolomic readouts (e.g., SCFAs) to identify responders and mechanistic pathways. Trials should also evaluate “real-world” implementation questions—dose and tolerability of fermentable fibers, optimal duration, and combined dietary patterns (e.g., MedDiet enriched with prebiotics and low-GI grains)—and assess safety signals relevant to insulin-treated populations, including delayed postprandial hypoglycemia. Ultimately, clarifying how nutrition can leverage preserved enteroendocrine function—alone or alongside immunomodulatory therapies—could help position dietary modulation of endogenous GLP-1 as a component of precision, stage-based T1D care.

## Figures and Tables

**Table 1:** Type 1 diabetes staging system (TrialNet/ADA criteria). Stages 1–2 (red arrows) represent the optimal window for incretin-modulating interventions due to preserved beta-cell mass (~30–80%) [6].

Stage	Autoantibodies	Glucose Metabolism	Beta-Cell Function	Clinical Status	Progression Risk
Stage 1	≥2 islet autoantibodies (GAD65, IA-2, ZnT8, IAA)	Normal (HbA1c < 5.7%, OGTT < 200 mg/dL)	~70–80% remaining	Asymptomatic	10–15% per year to Stage 2
Stage 2	≥2 islet autoantibodies persist	Dysglycemia (HbA1c 5.7–6.4% or abnormal OGTT)	~30–50% remaining	Asymptomatic	40–75% over 5 years to Stage 3
Stage 3	≥2 islet autoantibodies	Overt hyperglycemia (HbA1c ≥ 6.5%)	<10–20% (honeymoon phase possible)	Symptomatic, insulin required	Established T1D

**Table 2:** Summary of dietary interventions enhancing endogenous GLP-1 secretion in T1D and related conditions: mechanisms, evidence, and clinical recommendations.

Intervention	Key Mechanism	GLP-1 Effect	T1D Stage	Evidence in T1D/T2D	Optimal Dose/Duration	Key Studies
High-Fiber (β-glucan, inulin)	SCFAs → FFAR2/3 → L-cell Ca^2+^ influx	Mixed (↑ in animals; inconsistent human)	Stage 1–2 Priority	T1D: ↓ glycemic variability; C-peptide ↑	6–10 g/day, ≥6–12 weeks	[16,17,18,19,20,21,22,23,24,25]
Low-GI Carbs (e.g., isomaltulose)	Distal L-cell stimulation > proximal K-cell	↑ late GLP-1 AUC; ↓ GIP	Stage 1–2 Priority	T2D: improved glucose tolerance	GI < 55; acute/chronic	[26,27,28,29,30]
Mediterranean Diet	Unsaturated FAs (GPR120), polyphenols (cAMP/DPP-4), fiber	↑ biphasic GLP-1 AUC 15-40%	Stage 1–2, & 3	T2D: ↑ GLP-1 vs. vegetarian; endothelial protection	Holistic; olive oil focus	[31,32,33,34,35,36,37,38,39,40]
Low-Fat Vegan	↑ SCFA + ↓ GI + low-sat fat	Possible synergistic GLP-1 enhancement	Stage 3 Adjunct	T1D: RCT (n35)	12 weeks	[41]

## Data Availability

No new data were created or analyzed in this study. Data sharing is not applicable to this article.

## Abbreviations

The following abbreviations are used in this manuscript:

T1D	Type 1 diabetes
GLP-1	Glucagon-like peptide-1
GIP	Glucose-dependent insulinotropic polypeptide
SCFAs	Short-chain fatty acids
GI	Glycemic index
T2D	Type 2 diabetes
MedDiet	Mediterranean diet

## References

[1] Gasbjerg L.S., Christensen M.B., Hartmann B., Rosenkilde M.M., Holst J.J., Nauck M., Knop F.K. (2020). Evaluation of the incretin effect in humans using GIP and GLP-1 receptor antagonists. Peptides.

[2] Suen C.S., Burn P. (2012). The potential of incretin-based therapies in type 1 diabetes. Drug Discov. Today.

[3] Kielgast U., Holst J.J., Madsbad S. (2011). Antidiabetic actions of endogenous and exogenous GLP-1 in type 1 diabetic patients with and without residual β-cell function. Diabetes.

[4] Zhang L., Qin Y., Huang Y., Hu Q., Wu Q., Wang X., Zhang M. (2024). Abnormal late postprandial glucagon response in type 1 diabetes is a function of differences in stimulated C-peptide concentrations. Front. Endocrinol..

[5] Lawaetz T.W.H., Kücük Z., Andersen T.H., Jensen A.K., Johannesen J. (2025). Incretin hormone levels (GLP-1 and GIP) in children and adolescents with type 1 diabetes: A systematic review. Pediatr. Diabetes.

[6] Couper J.J., Haller M.J., Greenbaum C.J., Ziegler A.-G., Wherrett D.K., Knip M., Craig M.E. (2018). ISPAD clinical practice consensus guidelines 2018, stages of type 1 diabetes in children and adolescents. Pediatr. Diabetes.

[7] Herold K.C., Bundy B.N., Long S.A., Bluestone J.A., DiMeglio L.A., Dufort M., Gitelman S.E., Gottlieb P.A., Krischer J.P., Linsley P.S. (2019). An anti-CD3 antibody, teplizumab, in relatives at risk for type 1 diabetes. N. Engl. J. Med..

[8] Sperkowska B.M., Chrustek A., Gryn-Rynko A., Proszowska A. (2025). Dietary interventions for adults with type 1 diabetes: Clinical outcomes, guideline alignment, and research gaps—A scoping review. Nutrients.

[9] Cadario F. (2024). Insights in nutrition to optimize type 1 diabetes therapy. Nutrients.

[10] Nogal A., Valdes A.M., Menni C. (2021). The role of short-chain fatty acids in the interplay between gut microbiota and diet in cardio-metabolic health. Gut Microbes.

[11] Le Poul E., Loison C., Struyf S., Springael J.-Y., Lannoy V., Decobecq M.-E., Brezillon S., Dupriez V., Vassart G., Van Damme J. (2003). Functional characterization of human receptors for short chain fatty acids and their role in polymorphonuclear cell activation. J. Biol. Chem..

[12] Karaki S., Tazoe H., Hayashi H., Kashiwabara H., Tooyama K., Suzuki Y., Kuwahara A. (2008). Expression of the short-chain fatty acid receptor, GPR43, in the human colon. J. Mol. Histol..

[13] Tazoe H., Otomo Y., Karaki S., Kato I., Fukami Y., Terasaki M., Kuwahara A. (2009). Expression of short-chain fatty acid receptor GPR41 in the human colon. Biomed. Res..

[14] Tolhurst G., Heffron H., Lam Y.S., Parker H.E., Habib A.M., Diakogiannaki E., Cameron J., Grosse J., Reimann F., Gribble F.M. (2012). Short-chain fatty acids stimulate glucagon-like peptide-1 secretion via the G-protein-coupled receptor FFAR2. Diabetes.

[15] Christiansen C.B., Gabe M.B.N., Svendsen B., Dragsted L.O., Rosenkilde M.M., Holst J.J. (2018). The impact of short-chain fatty acids on GLP-1 and PYY secretion from the isolated perfused rat colon. Am. J. Physiol. Gastrointest. Liver Physiol..

[16] Zaremba S.M.M., Gow I.F., Drummond S., McCluskey J.T., Steinert R.E. (2018). Effects of oat β-glucan consumption at breakfast on ad libitum eating, appetite, glycemia, insulinemia and GLP-1 concentrations in healthy subjects. Appetite.

[17] Pino J., Marcos V., Amengual-Carbo J. (2021). Effect of dietary supplementation with oat beta-glucan for 3 months in subjects with type 2 diabetes: A randomized, double-blind, controlled clinical trial. J. Med. Food.

[18] Chen V., Zurbau A., Ahmed A., A Khan T., Au-Yeung F., Chiavaroli L., Mejia S.B., A Leiter L., A Jenkins D.J., Kendall C.W.C. (2022). Effect of oats and oat β-glucan on glycemic control in diabetes: A systematic review and meta-analysis of randomized controlled trials. BMJ Open Diabetes Res. Care.

[19] Bozbulut R., Şanlıer N., Döğer E., Bideci A., Çamurdan O., Cinaz P. (2020). The effect of beta-glucan supplementation on glycemic control and variability in adolescents with type 1 diabetes mellitus. Diabetes Res. Clin. Pract..

[20] Zhang Q., Yu H., Xiao X., Hu L., Xin F., Yu X. (2018). Inulin-type fructan improves diabetic phenotype and gut microbiota profiles in rats. PeerJ.

[21] Kietsiriroje N., Kanjanahirun K., Kwankaew J., Ponrak R., Soonthornpun S. (2018). Phytosterols and inulin-enriched soymilk increases glucagon-like peptide-1 secretion in healthy men: Double-blind randomized controlled trial, subgroup study. BMC Res. Notes.

[22] Steinert R.E., Mueller M., Serra M., Lehner-Sigrist S., Frost G., Gero D., Gerber P.A., Bueter M. (2024). Effect of inulin on breath hydrogen, postprandial glycemia, gut hormone release, and appetite perception in RYGB patients: A prospective, randomized, cross-over pilot study. Nutr. Diabetes.

[23] Birkeland E., Gharagozlian S., Gulseth H.L., Birkeland K.I., Hartmann B., Holst J.J., Holst R., Aas A.M. (2021). Effects of prebiotics on postprandial GLP-1, GLP-2 and glucose regulation in patients with type 2 diabetes: A randomised, double-blind, placebo-controlled crossover trial. Diabet. Med..

[24] Wang L., Yang H., Huang H., Zhang C., Zuo H.X., Xu P., Niu Y.M., Wu S.S. (2019). Inulin-type fructans supplementation improves glycemic control for the prediabetes and type 2 diabetes populations: Results from a GRADE-assessed systematic review and dose-response meta-analysis of 33 randomized controlled trials. J. Transl. Med..

[25] Ho J., Nicolucci A.C., Virtanen H., Schick A., Meddings J., Reimer R.A., Huang C. (2019). Effect of prebiotic on microbiota, intestinal permeability, and glycemic control in children with type 1 diabetes. J. Clin. Endocrinol. Metab..

[26] Esfahani A., Wong J.M., Mirrahimi A., Srichaikul K., Jenkins D.J., Kendall C.W. (2009). The glycemic index: Physiological significance. J. Am. Coll. Nutr..

[27] Salvatore T., Nevola R., Pafundi P.C., Monaco L., Ricozzi C., Imbriani S., Rinaldi L., Sasso F.C. (2019). Incretin hormones: The link between glycemic index and cardiometabolic diseases. Nutrients.

[28] Pfeiffer A.F.H., Keyhani-Nejad F. (2018). High glycemic index metabolic damage—A pivotal role of GIP and GLP-1. Trends Endocrinol. Metab..

[29] Kawai K., Okuda Y., Yamashita K. (1985). Changes in blood glucose and insulin after an oral palatinose administration in normal subjects. Endocrinol. Jpn..

[30] Keyhani-Nejad F., Kemper M., Schueler R., Pivovarova O., Rudovich N., Pfeiffer A.F. (2016). Effects of palatinose and sucrose intake on glucose metabolism and incretin secretion in subjects with type 2 diabetes. Diabetes Care.

[31] Rocca A.S., Brubaker P.L. (1995). Stereospecific effects of fatty acids on proglucagon-derived peptide secretion in fetal rat intestinal cultures. Endocrinology.

[32] Feltrin K.L., Little T.J., Meyer J.H., Horowitz M., Smout A.J.P.M., Wishart J., Pilichiewicz A.N., Rades T., Chapman I.M., Feinle-Bisset C. (2004). Effects of intraduodenal fatty acids on appetite, antropyloroduodenal motility, and plasma CCK and GLP-1 in humans vary with their chain length. Am. J. Physiol. Regul. Integr. Comp. Physiol..

[33] Ruiz-Pozo V.A., Guevara-Ramírez P., Paz-Cruz E., Tamayo-Trujillo R., Cadena-Ullauri S., Frias-Toral E., Simancas-Racines D., Altuna-Roshkova Y., Reytor-González C., Zambrano A.K. (2024). The role of the Mediterranean diet in prediabetes management and prevention: A review of molecular mechanisms and clinical outcomes. Food Agric. Immunol..

[34] Domínguez Avila J.A., Rodrigo García J., González Aguilar G.A., de la Rosa L.A. (2017). The antidiabetic mechanisms of polyphenols related to increased glucagon-like peptide-1 (GLP1) and insulin signaling. Molecules.

[35] Wang Y., Shen Y., Lu S., Wu J. (2023). EVOO supplement prevents type 1 diabetes by modulating gut microbiota and serum metabolites in NOD mice. Life Sci..

[36] Arukha A.P., Nayak S., Swain D.M. (2025). Effect of *Akkermansia muciniphila* on GLP-1 and insulin secretion. Nutrients.

[37] Bartimoccia S., Cammisotto V., Nocella C., Del Ben M., D’amico A., Castellani V., Baratta F., Pignatelli P., Loffredo L., Violi F. (2022). Extra virgin olive oil reduces gut permeability and metabolic endotoxemia in diabetic patients. Nutrients.

[38] Carnevale R., Loffredo L., Del Ben M., Angelico F., Nocella C., Petruccioli A., Bartimoccia S., Monticolo R., Cava E., Violi F. (2017). Extra virgin olive oil improves post-prandial glycemic and lipid profile in patients with impaired fasting glucose. Clin. Nutr..

[39] Ceriello A., Esposito K., La Sala L., Pujadas G., De Nigris V., Testa R., Bucciarelli L., Rondinelli M., Genovese S. (2014). The protective effect of the Mediterranean diet on endothelial resistance to GLP-1 in type 2 diabetes: A preliminary report. Cardiovasc. Diabetol..

[40] Bozzetto L., Alderisio A., Clemente G., Giorgini M., Barone F., Griffo E., Costabile G., Vetrani C., Cipriano P., Giacco A. (2019). Gastrointestinal effects of extra-virgin olive oil associated with lower postprandial glycemia in type 1 diabetes. Clin. Nutr..

[41] Kahleova H., Znayenko-Miller T., Smith K., Khambatta C., Barbaro R., Sutton M., Holtz D.N., Sklar M., Pineda D., Holubkov R. (2024). Effect of a dietary intervention on insulin requirements and glycemic control in type 1 diabetes: A 12-week randomized clinical trial. Clin. Diabetes.

